# Determinants of patient-reported experience of cancer services responsiveness

**DOI:** 10.1186/s12913-015-1104-9

**Published:** 2015-09-28

**Authors:** Dominique Tremblay, Danièle Roberge, Djamal Berbiche

**Affiliations:** Nursing School, Faculty of Medicine and Health Sciences, Université de Sherbrooke, Longueuil, QC Canada; Charles-Le Moyne Hospital Research Centre, Greenfield Park, Longueuil, QC Canada; Community Health Sciences Department, Faculty of Medicine and Health Sciences, Université de Sherbrooke, Longueuil, QC Canada

**Keywords:** Responsiveness, Cancer, Patient-reported experience, Ambulatory oncology

## Abstract

**Background:**

In coming years, patient-reported data are expected to play a more prominent role in ensuring early and efficient detection of healthcare system dysfunctions, developing interventions and evaluating their effects on health outcomes, and monitoring quality of care from the patient’s perspective. The concept of responsiveness relates to patient-reported experience measures that focus on the system’s response to service users’ legitimate expectations. We explored this concept in an effort to address unresolved issues related to measuring and interpreting patient experience. Our objectives in this study were to report on patients’ perceptions of cancer services responsiveness and to identify patient characteristics and organizational attributes that are potential determinants of a positive patient-reported experience.

**Methods:**

A cross-sectional survey was conducted of 1379 cancer patients in nine participating ambulatory cancer clinics in hospitals across the province of Quebec, Canada. They were invited to complete the Cancer Services Responsiveness tool, a 19-item questionnaire evaluating patients’ perceptions of the responsiveness of cancer services. Sociodemographic data and self-reported clinical and organizational data were collected. Descriptive statistical analysis, univariate and multivariate logistic regressions were performed.

**Results:**

The patients surveyed generally perceived cancer services as highly responsive. The individual determinants of overall responsiveness found to be significant were self-assessed health status, age, and education level; organizational determinants were academic affiliation and geographic location of the clinic.

**Discussion:**

Responsiveness refers to distinctive indicators of healthcare quality focused on patient-provider interactions and presents a complementary picture to other patient-reported experience measures. The identified determinants of patients’ positive experience with cancer services provide valuable information to guide care providers in targeting quality improvements.

**Conclusions:**

Finally, our results suggest these determinants should be further studied to eliminate confounders and produce usable results.

**Electronic supplementary material:**

The online version of this article (doi:10.1186/s12913-015-1104-9) contains supplementary material, which is available to authorized users.

## Background

Patient-reported experience data that capture patients’ views of what happened during care processes are metrics that are increasingly recognized as usable data to improve patient-provider relationships and to evaluate the quality of care delivery [1]. In the oncology sector, patient-reported data are recommended [2], as they enable health system actors to develop a broader understanding of care quality. The two main domains advocated for capturing patients’ perspectives on the effects of cancer and its treatment are: 1) patient-reported outcome measures (PROMs), which measure the impact of an illness and the effects of interventions on symptomatology and well-being (e.g. physical function, emotional distress, health-related quality of life, health status) [3, 4]; and 2) patient-reported experience measures (PREMs), which capture patients’ views of what happened during the health encounter (care processes) [5, 6].

Responsiveness relates to PREMs. The aim of collecting patient-reported experiences of responsiveness is to learn whether care processes are responsive to patients’ needs and well-being. However, the research community has yet to resolve certain issues related to the measurement and interpretation of patient experience, such as choice of survey content, potential confounders, and the mode and timing of questionnaire administration. The challenge is to develop a more robust corpus of knowledge without overwhelming service users along the way with cumbersome instruments and processes. If improvements in health outcomes are to be achieved by using PREMs in clinical and health system decision-making, efforts must be deployed to meet this challenge.

The 2000 World Health Report [7] has paved the way for PREMs, putting service users’ legitimate expectations at the forefront of health systems responsiveness. According to Valentine and colleagues [8], responsiveness refers to the manner and environment in which people are treated when they interact with the healthcare system or one of its components. The original responsiveness concept focused on eight aspects of patient-reported experience, selected for their relevance to health systems performance: autonomy, choice, communication, confidentiality, dignity, prompt attention, quality of basic amenities, and support (access to family and community support networks). Responsiveness relates specifically to the interactional dimensions of patient experience rather than to health-related or technical aspects of care quality.

The World Health Organization (WHO) responsiveness instrument was originally used to evaluate and compare health systems in countries with different characteristics such as gross domestic product, income per capita, and expenditure per capita. At the country level, the WHO responsiveness instrument has been used to compare patient perceived health services responsiveness in Organisation for Economic Co-operation and Development countries and developing countries [8, 9]. The instrument has also been used in a few studies to measure responsiveness among specific clienteles, such as patients with mental health problems [10], human immunodeficiency virus [11], or cancer [12]. In these studies, the clinical or socio demographic factors were used to describe the sample and not to investigate the individual or organizational determinants of health services responsiveness. Further, while evidence on clinical and sociodemographic determinants of perceived quality of cancer care has accumulated over the last decade [5, 13], evidence for organizational determinants of patient perception of the quality of cancer care is still lacking [14, 15]. According to Zapka and colleagues, intensive investigations of a combination of potential determinants of organizational, provider, and patient characteristics are required to improve quality of cancer care [16]. Our study represents an effort to fill these knowledge gaps.

With a view to furthering the development of PREMs and fostering their use in the oncology sector, our objectives in this study were: 1) to report on patient-reported experience of cancer services responsiveness (CSR), and 2) to identify the patient characteristics and organizational attributes that are potential determinants of a positive patient-reported experience.

## Methods

### Study setting

The study was conducted in Quebec, Canada. This province has a publicly funded healthcare system providing universal access to medical services for over eight million residents. The province’s cancer plan was launched in 1998 to enhance the accessibility, coordination, continuity, and responsiveness of patient-centred care. The Regional Health Authorities have been responsible for implementing the key elements of that plan. The strengthening of interdisciplinary functioning in cancer teams, the introduction of more than 250 nurse navigators in oncology (also called pivot nurse or nurse coordinator), and the implementation of integrated cancer networks are illustrations of efforts invested in translating the national cancer plan into practice and improving CSR [17].

### Study design and procedures

We conducted a cross-sectional survey of cancer patients attending the ambulatory cancer clinics of nine participating hospitals across the province of Quebec in 2011. This sample represents 15 % of all hospitals in Quebec providing cancer care. The hospitals were purposefully selected to represent the diversity of cancer clinics with a variety of organizational attributes that are presumed to influence care delivery and ultimately CSR (Table 1). Eligible patients were adults of 18 years of age and over, had a confirmed cancer diagnosis (all cancers, all stages), had visited the ambulatory cancer clinic at least once in the preceding 12 months, and read and understood either French or English.

**Table 1:** Organizational attributes of, and participants from, participating sites (*N* = 9)

Site	Mandate^a^	Academic affiliation	Cancer team size^b^	Geographic location	Participants^c^	
					N	(%)
1	Regional	Yes	Small	Rural	158	(11.5)
2	Regional	Yes	Large	Urban	202	(14.6)
3	Regional	Yes	Large	Semi-rural	158	(11.5)
4	Local	No	Small	Rural	98	(7.1)
5	Local	No	Small	Rural	86	(6.2)
6	Regional	No	Large	Semi-rural	140	(10.2)
7	Local	Yes	Small	Rural	143	(10.4)
8	Local	Yes	Small	Urban	214	(15.5)
9	Local	No	Large	Urban	180	(13.1)
Total					1379	(100)

^a^At the time of the study

^b^Cancer team with 8 professionals from different disciplines and more = large; Fewer than 8 = small

^c^Participants with completed questionnaires included for the statistical analysis

All cancer patients were recruited consecutively upon arrival at the clinic, whether for treatment, a medical consultation, or a follow-up visit. Designated staff members were trained to identify potential participants and to provide them with relevant information about the study in a systematic manner. Patients who agreed to participate were given a cover letter, the self-administered survey questionnaire, and a return envelope. A reminder was mailed 2 weeks after initial distribution of the questionnaire to optimize response rate [18]. Participation in the survey was voluntary and anonymous. The cover letter was provided to the participants in order to explain inform consent. This letter included the project description, the participation involvement and ethical issues. The action of answering anonymously to the questionnaire and to post it back to the research team was considered as a consent to participate to the study. This procedure and the entire study were approved by the Research Ethics Board of the Charles-Le Moyne Hospital Research Centre (ref. number MP-HCLM-09-050).

### Patient survey questionnaires

The CSR questionnaire comprised 19 items grouped into four subscales: prompt access to care (PAC), person-centred response (PCR), quality of patient-provider communication (COM), and quality of care environment (QCE) (see Additional file 1: Table S1). The item formulations were adapted for Quebec cancer services from WHO’s generic responsiveness instrument [8]. They were translated into French, and a validation study of the adapted French version was performed [12]. A four-point Likert-type scale (1 = never to 4 = always) was used for all the items. Respondents were asked to evaluate CSR for services provided within the preceding 12 months. The questionnaire was available in French and English.

Additional data were collected to identify variables that were potential determinants of responsiveness. From the literature on patient satisfaction and quality of care, we selected 15 variables based on their potential effects on perceived experience of care by cancer patients and other clienteles with chronic diseases [5, 13, 19, 20]. The sociodemographic characteristics included were age, gender, level of education completed, self-assessed health status, perceived financial situation, and perceived emotional well-being.

Variables related to participants’ clinical characteristics at time of recruitment were: time since diagnosis, whether consulting for a new cancer or a relapse of a previously treated cancer, cancer site, types of treatment received in the preceding 12 months, and comorbidity.

Four potential organizational attributes were identified in the literature on patient satisfaction and quality of care as being more critical: specialization based on the hospital’s mandate, academic affiliation, geographic location, and cancer interdisciplinary team size and diversity [14, 21–26]. Detailed descriptions of these variables are provided in Additional file 1.

### Statistical analyses

Statistical analyses were performed using IBM SPSS Statistics for Windows, Version 19.0 and SAS 9.2 for Windows. To examine patient-reported experience of CSR (Objective 1), data were scored according to the four CSR subscales—PAS, COM, PCC, QPE—and the overall CSR. Internal reliability was tested using Cronbach’s alpha.

Because experienced responsiveness was high and the distribution was skewed toward higher values, the variables were dichotomized, so that respondents providing positive responses (always or often) for more than 75 % of the subscale items were classified as having a positive experience. Binary variables were created: positive patient reported experience = 1, less positive patient reported experience = 2 [27].

Following Spearman analysis, variables significantly correlated with the overall responsiveness scores were included in the regression analysis to identify determinants of overall CSR and each of the four subscales (PAS, COM, PCC, QPE). Univariate logistic regression analyses were performed to screen a subset of variables among patient characteristics and organizational attributes. The variables retained in the univariate screening were then used in a multivariate regression analysis to identify determinants of perceived responsiveness (Objective 2). Statistical significance was assumed at *p* <0.05 for all tests.

## Results

### Response rate and baseline patient characteristics

**Fig. 1:**
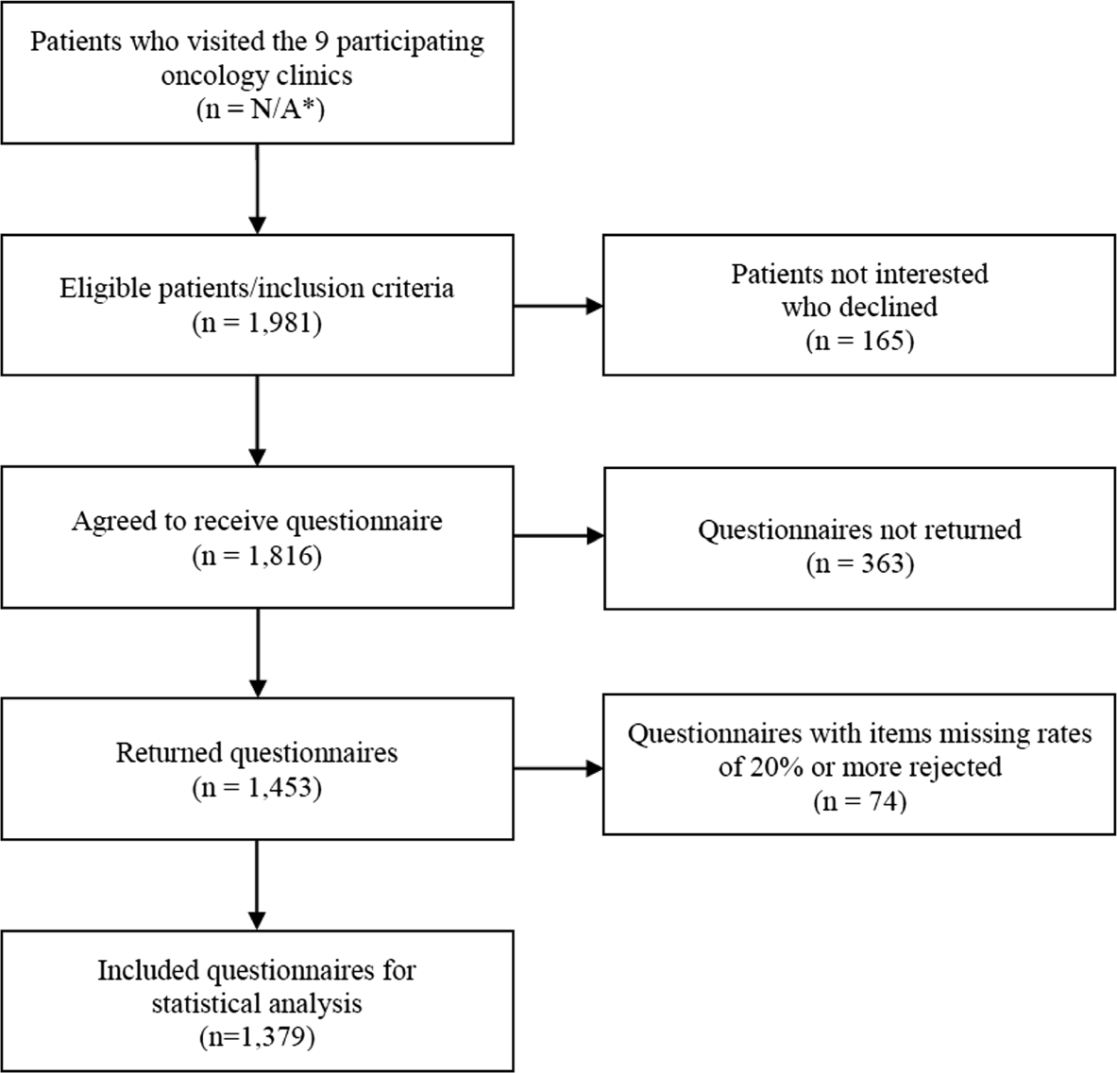
Patient data flow-chart N/A: The information is not available. The method used to calculate the volume of activity is not the same for each clinic

Between October 2010 and November 2011, a total of 1981 outpatients in the nine clinics met our inclusion criteria (Fig. 1). Of these, 165 (8 %) patients declined to participate, 1816 agreed to receive the questionnaire, and 1453 (73.3 %) returned it by mail. Returned questionnaires with items missing rates of 20 % or more were rejected, leaving 1379 to be included for statistical analysis.

Table 2 displays the patients’ baseline sociodemographic and clinical characteristics. The mean age of our study population was 61 years (SD = 11), with a majority in the 50–69 age group. A preponderant proportion of respondents were women (61.9 %); 37.3 % of respondents had higher education levels, 57 % felt they earned enough money and 21.9 % were financially comfortable. Half of the respondents reported having good health status. Breast cancer and colorectal cancer were the most frequent cancer types found in our study population, with nearly one-quarter reporting a relapse. Near 56 % were within the first year since diagnosis and a great majority had received chemotherapy, either alone or in combination with another type of cancer treatment. Finally, 34.3 % reported having no comorbidity.

**Table 2:** Patient sociodemographic and clinical characteristics (*N* = 1379)

Characteristics		Percent	Number^a^
Sex			
Female		61.9	845
Age			
Mean age (SD)	61 (SD 11)		
18–49 years		15.7	214
50–69 years		61.5	839
70 years and over		22.9	312
Education level (completed)			
Primary		18.3	246
Secondary		44.4	598
Business college/CEGEP^b^		15.7	211
University		21.6	291
Perceived financial status			
Financially comfortable		21.9	291
Earn enough		57.0	757
Poor		18.8	249
Very poor		2.3	31
Cancer type			
Breast		26.5	359
Colorectal		21.4	290
Hematopoietic		15.9	216
Bronchopulmonary		14.2	192
Female genital		4.6	62
Other		17.5	238
Time since diagnosis (years)			
< 1		55.7	758
1 to 3		27.7	377
≥ 3		16.7	227
Treatment type			
Chemotherapy only		39.0	519
Chemotherapy + other treatment		49.1	653
Other		6.8	91
None		5.1	68
Self-assessed health status			
Good		50.4	683
Poor		49.6	672
Comorbidities			
0		34.3	473
1 to 3		59.4	819
More than 3		6.3	87
Emotional well-being			
Good		47.7	647
Poor		52.3	708

^a^Total n may vary per characteristic due to missing values

^b^In Quebec, business colleges and CEGEPs are post-secondary institutions that provide pre-university education (2 years) or specialized vocational programs (3 years)

### Cancer services responsiveness

Table 3 presents the Cronbach’s alpha and mean scores with standard deviations (SD) for each subscale and for overall CSR. The reliability for overall responsiveness was 0.90 and ranged from 0.64 to 0.85 for subscales. The overall responsiveness mean score was 3.63 (SD 0.16). Mean subscale scores ranged from 3.34 (SD 0.69) to 3.77 (SD 0.41) for prompt access to care and person-centred response, respectively.

**Table 3:** Dimensions of CSR, mean score and standard deviation (SD)

Dimension of CSR and subscales^a^	Alpha	Number of items	Mean score^b^	SD^c^
Prompt access to care (PAC)	0.77	4	3.34	0.69
Person-centred response (PCR)	0.67	5	3.77	0.41
Patient-provider communication (COM)	0.85	5	3.61	0.56
Quality of care environment (QCE)	0.64	5	3.72	0.37
Cancer services responsiveness (CSR)	0.90	19	3.63	0.16

^a^According to the CSR validation study [12]

^b^The Likert-type scale ranged from 1 to 4

^c^Standard deviation

### Factors associated significantly with CSR

Table 4 displays the results of multivariate logistic regression analysis on the effects of individual and organizational factors on CSR. Patients with good self-assessed health status were more likely to report positive overall responsiveness (OR = 1.64, [1.28–2.10]) than those with poor self-assessed health status. Patients 70 years of age and over were 1.73 times [1.17–2.56] more likely than younger patients to rate overall responsiveness favourably. Compared to patients with higher education levels, those with less education were 1.43 times [1.12–1.83] more likely to rate overall responsiveness positively. Finally, gender and perceived emotional well-being did not seem to influence perceived responsiveness when all variables were entered into the model. The patient characteristics associated with most of the CSR subscales were self-assessed health status and level of education. The results of the univariate logistic regression analysis are described in Additional file 2.

**Table 4:** Multivariate multilevel logistic regression of patient and organizational determinants of cancer services responsiveness

		Patient reported experience measure				
		PAC	PCR	COM	QCE	CSR
		*n* = 567/563	*n* = 949/343	*n* = 833/460	*n* = 965/329	*n* = 805/491
		OR [CI 95 %]	OR [CI 95 %]	OR [CI 95 %]	OR [CI 95 %]	OR [CI 95 %]
Patient characteristics						
Self-assessed health status^a^	Good	1.29	1.95*	1.61*	1.54*	1.64*
		[0.96–1.73]	[1.48–2.57]	[1.25–2.07]	[1.16–2.03]	[1.28–2.10]
Age^b^	50–69	1.15	0.99	1.25	1.55*	1.26
		[0.78–1.69]	[0.70–1.42]	[0.9–1.73]	[1.09–2.19]	[0.91–1.74]
	70 years and over	1.33	1.34	1.69*	3.07*	1.73*
		[0.83–2.11]	[0.87–2.06]	[1.13–2.51]	[1.95–4.83]	[1.17–2.56]
Gender^c^	Men	1.37*	1.23	1.02	1.11	1.19
		[1.031.81]	[0.94–1.62]	[0.79–1.31]	[0.84–1.47]	[0.94–1.53]
Education^d^	High school and less	1.19	1.52*	1.65*	1.31*	1.43*
		[0.89–1.61]	[1.17–1.99]	[1.28–2.11]	[0.99–1.72]	[1.12–1.83]
Emotional well-being^e^	Good	1.44*	1.07	1.14	1.47*	1.18
		[1.07–1.95]	[0.81–1.41]	[0.88–1.47]	[1.11–1.95]	[0.92–1.52]
Organizational attributes						
Mandate^f^	Local	0.81	1.10	0.97	0.92	1.01
		[0.51–1.30]	[0.71–1.70]	[0.65–1.46]	[0.59–1.45]	[0.68–1.50]
Academic affiliation^g^	Yes	0.89	0.92	0.75	0.53*	0.69*
		[0.60–1.34]	[0.63–1.33]	[0.53–1.05]	[0.37–0.78]	[0.49–0.97]
Team size^h^	Large	0.69	1.76*	1.34	0.60	1.07
		[0.38–1.27]	[1.03–3.02]	[0.81–2.20]	[0.34–1.06]	[0.66–1.76]
Geographic location^i^	Semi-rural	1.01	1.00	0.89	0.80	1.09
		[0.64–1.61]	[0.66–1.52]	[0.61–1.30]	[0.54–1.19]	[0.75–1.59]
	Rural	1.80*	1.65*	1.81*	1.14	1.74*
		[1.07–3.02]	[1.05–2.61]	[1.18–2.78]	[0.70–1.86]	[1.13–2.65]

Results show odds ratios (OR) and confidence intervals [CI] 95 % when both patient characteristics and organizational attributes are in the model

*p < 0,05

^a^Reference category (1) = Bad

^b^Reference category (1) = 18–49

^c^Reference category (1) = Women

^d^Reference category (1) = College and more

^e^Reference category (1) = Negative perception

^f^Reference category (1) = Regional

^g^Reference category (1) = No

^h^Reference category (1) = Small < 8

^i^Reference category (1) = Urban

Regarding organizational attributes, two variables were deemed significantly associated with overall CSR. Patients treated in rural clinics were 1.74 times more likely than those in urban areas to rate CSR positively. Geographic location was also found to be a factor influencing most of the CSR subscales. Compared to patients treated in clinics with no academic affiliation, those followed in clinics with academic affiliation tended to rate both overall responsiveness (OR = 0.69; [0.49–0.97]) and the quality of the environment (OR = 0.53; [0.37–0.78]) less positively.

## Discussion

### Key findings

This study examined patient-reported experience of CSR in Quebec and identified individual and organizational determinants of positive patient experience. With regard to our first objective, the key finding was that patient-reported experience of CSR was largely positive, with very little variation in our sample. PAC was the least positive aspect of the patients’ care experience. Regarding the second objective, a final explicative model emerged that included six determinants of a positive CSR experience. These determinants are: self-assessed health status (good), age (70 years and over), education (lower levels), emotional well-being (good), geographic location (rural), and academic affiliation of hospital (no affiliation). From our explicative model, a predominant pattern of determinants emerged related to overall responsiveness, but different explanatory models appeared when considering responsiveness subscales. These results suggest that individual and organizational factors may have differential effects on different aspects of responsiveness, such that efforts to improve services will require multiple interrelated interventions addressing both individual and organizational dimensions. Nevertheless, good self-assessed health status, lower education levels, and receiving services in a rural location are more likely to be associated with a positive patient experience.

Our findings are consistent with related studies and reports on the quality of cancer services. Positive perceptions of CSR may be the result of improvement strategies implemented over the past decade, at both the national and local levels, to encourage a comprehensive patient-centred approach, better care coordination through the deployment of cancer nurse navigators (also called oncology pivot nurses in Quebec), and stronger interdisciplinary cancer teams [28].

Prompt access to care (PAC) is a growing problem in many developed countries, and Quebec is no exception. In our study, PAC referred to wait times before a scheduled consultation and the patient’s capacity to reach a professional when unanticipated health problems arose outside of clinic hours, including weekends. Excessive time spent in the waiting room before a consultation has been identified elsewhere as a source of overall dissatisfaction among patients with cancer [29]. Access to care for unanticipated health problems related to cancer may be associated with both patient characteristics and organizational attributes [30]. Recent literature has demonstrated that, even within a publicly funded health system, access to free health services does not necessarily ensure equality of access for patients with different socioeconomic characteristics. Our results showed that male gender and self-reported good emotional well-being were associated with a more positive perception of PAC.

Our explanatory model revealed a predominant pattern of determinants for CSR, but also that the influencing factors differed depending on the responsiveness dimensions. This result suggests that patient characteristics and organizational attributes may have different effects on responsiveness dimensions. The effects are described in greater detail below.

Overall CSR is predicted by patient characteristics such as perceived self-assessed health status, age, gender, education, and emotional well-being. In this study, emotional well-being was captured using questions on emotional distress and anxiety, negative affect, and psychological adjustment to the disease. This may be an area for service improvement, since distress has been described as the sixth vital sign for cancer patients [31]. Heightened distress has been associated with worse patient outcomes, in terms of poorer health-related quality of life, reduced adherence to treatment, decreased satisfaction with care, and possibly lower survival rates. Our study highlights the importance of planning psychosocial interventions to improve patients’ perceived emotional well-being that have the potential to influence other dimensions of their healthcare experience as well.

We identified organizational determinants of CSR. Geographic location of the cancer clinics was the most consistent organizational determinant, expressed as higher levels of CSR in rural regions. This was surprising, given the prevailing assumption that, as specialized cancer services are located in urban hospitals, cancer patients and their families in non-metropolitan regions would view services less positively due to limited accessibility of cancer care infrastructures and resources and the travel distances involved. Empirical research has demonstrated a link between rural geographic location and access inequities along the cancer trajectory, from screening to end of life or survival, but with the exception of follow-up care, for which no such link has yet been demonstrated. Lamarche et al. (2010) suggest that certain characteristics of rural settings, such as community cohesion, may have a positive influence on components of the patient care experience, particularly responsiveness [32].

Our study revealed variations in patients’ experiences in hospitals with academic affiliations. Patients in those settings were less likely to report a positive perception of patient-centred care and overall responsiveness. There are many characteristics differentiating academic and community hospitals that may influence responsiveness. The academic mission is complicated by the large number of trainees dividing their time among clinical, academic, and research activities. It may be that these competing obligations reduce the number of person-centred practices, leading to a less positive perception among patients of professionals’ willingness to listen to their preferences and values [33, 34].

Nevertheless, our study showed that patients receiving care from larger cancer teams with professionals from different disciplines were more likely to have a positive perception of person-centred response. Several studies have demonstrated that multidisciplinary care models are generally associated with a more positive patient experience. These teams mobilize a variety of expertise to address health and wellness needs across the continuum of cancer care and to develop interdisciplinary care plans that take into account the whole person and family in their specific circumstances. However, there is no clear consensus as yet on the appropriate number and diversity of professionals in interdisciplinary teams [35–38]. Moreover, the internal functioning of interdisciplinary teams is understudied due to a lack of valid instruments to examine the greater diversity of professionals now found in teams in a variety of settings, such as the Quebec cancer teams [39, 40]. Finally, our study showed that patients treated in academic settings were less likely to report positive overall responsiveness. Another study has also reported that cancer patients treated in non-academic settings reported higher overall satisfaction compared to patients treated in academic affiliated hospitals [33].

### Unanswered questions and future research

Numerous studies have shown that cancer patients are generally satisfied with outpatient [41, 42] and in-hospital cancer care. Fewer studies have focused on PREMs such as CSR, even though it is recognized as a key element of the national cancer services plans in several countries. The results of our study offer a complementary perspective to patient satisfaction studies. They provide a picture of how a person-centred response may be achieved in an improved interdisciplinary care structure, such as that promoted by Quebec’s cancer program.

### Strengths and limitations of the study

To the best of our knowledge, this is the first study to specifically report on perceived CSR as a PREM, as well as on its determinants. This study is original, in that we explored explicative models of the determinants of CSR using not only overall perceived responsiveness but also each of its subscales. This approach has produced a more detailed picture of patient-reported experience. There is a distinction to be made between patients’ own reports of what actually happened (or not) and their levels of satisfaction with the healthcare system [39, 40]. The literature highlights the difficulties of measuring patient satisfaction for use in improving patient care and rarely provides specific information that can be acted upon to achieve change. Nor are published studies reliable in capturing multiple care events and multiple interactions over time, as occurs in the cancer trajectory. Moreover, satisfaction can be influenced by a combination of elements not related to direct experience of services, such as personal expectations, perceived needs, and disease characteristics [4, 39]. Disentangling these influences can be difficult. PREMs, such as CSR, have been developed to be easier to interpret and provide more practical results. Satisfaction measures will continue to provide one facet of the patient experience, but monitoring CSR may offer a complementary perspective on the patient experience.

This study’s originality lies also in the fact that it identified not only clinical and demographic determinants of CSR, but also included organizational attributes in the analysis models. We believe it is important to take into account the effects of specific organizational variables, because patient experience is embedded in context, which can be acted upon to improve services. It is relatively straightforward to collect a huge quantity of data on patients’ views and opinions, but this data needs to be carefully contextualized to produce information that can be useful in setting targets for improvement.

Our results clearly support the idea that improvement programs should not only focus on clinical aspects of care at the practice level, but also include strategies to overcome organizational models that compromise responsive care [16]. Unfortunately, the current cancer system directs its resources largely toward addressing the former problems and often ignores the latter. Finally, one of the strengths of this study was the constitution of a real-world representative sample of patients in the cancer clinics in Quebec region. Given the high response rate (73.3 %) among patients and the selection of clinical settings with a variety of organizational attributes, we are reasonably confident the results of this study can be generalized to outpatients receiving care in cancer clinics with similar organizational context [43]. The principal study limitation involves the sampling method used in the participating ambulatory cancer clinics. Despite explicit patient selection criteria and a formal recruitment scenario to limit selection bias, it is possible that hospital staff recruited patients who were more positive about their care. If so, this selection bias could have contributed to the observed high responsiveness scores. Assuming that non-participants are similar to the study population, we used data available from a national survey evaluating the quality of cancer care in Québec (*n* = 9175) [44]. We used participant characteristics (sex and mean age) to compare non-participants to participants. Age and gender distribution of both the study sample and the non-participant sample were similar with mean age being respectively 61.10 years (SD = 11.73) and 62.93 years (SD = 12.66), *t*-value = 1.88, *p*-value of 0.06 and gender distribution, 38.10 and 45.45 % males, *χ*^2^ = 3.3547, *p*-value of 0.07. So there was no statistical difference between both samples for these two characteristics. Given the available information from the national survey, comparisons for other characteristics were not possible suggesting that one should be cautious with the generalizability of our study findings [45]. Additionally, considering the universal access of medical services in Quebec, our results may not be entirely applicable to other healthcare systems where access to medical services is not universal. Nevertheless, the analysis approach used and the strategies to reduce potential bias, leads us to be relatively confident in the conclusions that can be drawn from this study. Finally, our results suggest that rural location of the clinic was the most consistent organizational determinant of a positive patient perception of CSR. Since our definition of rural excluded remote regions of the province which may have different dynamics, this result should be interpreted with caution.

## Conclusions

CSR may be a useful PREM for describing pockets of excellence in cancer services as well as for identifying improvemen priorities around patient-centred care and patient-provider partnerships. Our study showed that cancer services in Quebec are responsive to patients in different aspects of their experience, such as quality of patient-provider communication, patient-centred response, and quality of care environment. Nevertheless, excellence in ensuring access to care remains a challenge, and efforts to reduce wait times and facilitate patient navigation through the system for ambulatory care require careful attention. These issues are of concern in all jurisdictions seeking to provide responsive care to patients and their families.

## Additional files

Additional file 1:
**Patient survey questionnaire items.** The questionnaire used in this study consisted of two sections. The first was designed to measure cancer services responsiveness from the patient’s perspective and the second to identify the main determinants of responsiveness. (DOC 96 kb) Additional file 2:
**Effects of patient characteristics and organizational attributes on cancer services responsiveness (univariate logistic regression).** Additional file 2 presents the results for the univariate logistic regression to identify the effects of patient characteristics and organizational attributes on cancer services responsiveness subscales: prompt access to care (PAC), person-centred response (PCR), quality of patient-provider communication (COM), and quality of care environment (QCE) and overall CSR. (DOCX 22 kb)

## Abbreviations

COM: Quality of patient-provider communication
CSR: Cancer services responsiveness
OR: Overall responsiveness
PAC: Prompt access to care
PCR: Person-centred response
PREMs: Patient-reported experience measures
PROMs: Patient-reported outcome measures
QCE: Quality of care environment
SD: Standard deviations
WHO: World Health Organization
