# Supplementary material for: Determinants of patient-reported experience of cancer services responsiveness
Source: BMC Health Serv Res. 2015 Sep 28;15:425. doi: 10.1186/s12913-015-1104-9 (PMC4587918; doi:10.1186/s12913-015-1104-9)
Supplement: Additional file 1: — Patient survey questionnaire items. The questionnaire used in this study consisted of two sections. The first was designed to measure cancer services responsiveness from the patient’s perspective and the second to identify the main determinants of responsiveness. (DOC 96 kb) [file 12913_2015_1104_MOESM1_ESM.doc]

# Additional file 1 - Patient survey questionnaire items

The questionnaire used in this study consisted of two sections. The first was designed to measure cancer services responsiveness from the patient’s perspective and the second to identify the main determinants of responsiveness.

### Section 1. Cancer services responsiveness questionnaire items

Cancer services responsiveness (CSR in the following text) comprised 19 questions grouped into four subscales, each referring to one dimension of the quality of care and services in oncology: prompt access to care (PAC), patient-centred response (PCR), quality of providers-patient communication (COM) and quality of the care environment (QCE) (Table 1).

### Section 2. Potential determinants questionnaire items

Additional data were collected to identify variables that were potential determinants of responsiveness (Table 2). Fifteen variables were selected based on their possible effects on perceived experience of care (satisfaction, quality of care) by cancer patients and other clienteles with chronic diseases . These variables relate to sociodemographic (six), clinical (four) and organizational (five) characteristics.

Included sociodemographic characteristics were: age, gender, level of education completed, self-assessed health status, perceived financial situation, and perceived emotional well-being. This last variable was a composite score of six items of the Emotional well-being subscale (6 items) from the Health Education Impact Questionnaire (heiQ) . Study patients were dichotomized into two groups based on their heiQ mean scores (above mean = good; below mean = poor). Variables characterizing patients’ clinical characteristics at the moment they were recruited into the study were: time since diagnosis and whether they were consulting for a new cancer or a relapse of a cancer treated in the past; cancer site treated (colorectal, female genital, prostate, hematopoietic, breast, head and neck, bronco-pulmonary, other); and treatment type received in the preceding 12 months. Comorbidity was assessed by a count of self-reported chronic diseases: diabetes mellitus, hypertension, coronary heart disease, cerebrovascular disease, lung disease, renal disease, arthritis, osteoarthritis, mental health disease. All patients’ characteristics were self-reported.

To identify possible organizational determinants, a review of the literature was conducted on patient satisfaction and quality of care from both the general and cancer sectors . Four potential organizational attributes were identified as more critical: 1) specialization based on the hospital’s mandate (local ‒ regional); 2) academic affiliation (yes ‒ no); 3) geographic location (urban ‒ semi-rural ‒ rural) based on population density and distance from highly specialized cancer centres ; and 4) cancer team size and diversity (cancer team with 8 professionals from different disciplines and more = large; fewer than 8 = small) .

## Table S1. Cancer services responsiveness questionnaire items

| **Item no. Question: In the past 12 months…:** | |  |
| --- | --- | --- |
|  |  |  |
| **Prompt access to care (PAC)** | |  |
| 1 | On weekdays it was possible to contact a professional at the cancer clinic by phone when needed |  |
| 2 | On weekdays it was possible to consult the cancer clinic on the same day in cases of emergency or complications |  |
| 3 | During evenings, at night and on weekends it was possible to contact a hospital professional by phone in cases of emergency or complications |  |
| 4 | The amount of time spent in the waiting room before a consultation was reasonable |  |
| **Patient-centred response (PCR)** | |  |
| 5 | Professionals provided you with the information you needed within a reasonable time given your health status |  |
| 6 | Professionals asked for your consent before beginning a treatment or test |  |
| 7 | Professionals addressed all your needs |  |
| 8 | The confidentiality of your personal information was respected |  |
| 9 | Your close relatives were encouraged to be present as much as you wanted |  |
| **Quality of provider–patient communication (COM)** | |  |
| 10 | Professionals listened to you attentively |  |
| 11 | Professionals gave you simple explanations that were easy to understand |  |
| 12 | Professionals provided you with enough time to ask all the questions you had about your illness and treatments |  |
| 13 | Professionals encouraged you to participate as much as you wanted in decisions regarding your care, treatment and tests |  |
| 14 | Professionals helped you assess the "pros" and "cons" regarding care, treatment and tests |  |
| **Quality of care environment (QCE)** | |  |
| 15 | Professionals treated you with respect |  |
| 16 | The waiting room was comfortable |  |
| 17 | The administrative staff treated you respectfully |  |
| 18 | Tests and treatments were conducted in a way that respected your physical privacy |  |
| 19 | You were able to speak privately with the professionals at the clinic |  |
| High values indicate high quality (4 = always). For items 24 to 28, low scores (1 = never) represent high quality. For comparative purposes, scores of items 24 to 28 were inversed. | | |

## Table S2. Potential determinants questionnaire items

| **Item no.** | **Variable** | | **Operationalization** |
| --- | --- | --- | --- |
| **Patients’ sociodemographic characteristics** | | |  |
| 1 | | Age (mean, SD) | 18–49 years; 50–69 years; 70 years and older |
| 2 | | Sex | Male; Female |
| 3 | | Level of education (completed) | Primary + Secondary;  Business college/CEGEP**+ University |
| 4 | | Self-assessed health status | Poor; Good |
| 5 | | Perceived financial status | Very poor + poor;  Earn enough +Financially comfortable |
| 6 | | Emotional well-being* | Poor; Good |
| **Patients’ clinical characteristics** | | | |
| 7 | | Time since diagnosis | < 1 year; 1–3 years; ≥ 3 years |
| 8 | | New cancer or relapse | New cancer; Relapse |
| 9 | | Cancer type | Colorectal, female genital, prostate, hematopoietic, breast, head and neck, broncho-pulmonary, other |
| 10 | | Treatment type (in the past 12 month) | Chemotherapy only; Chemotherapy + radiotherapy + surgery; Other; None |
| 11 | | Comorbidities | No comorbidity; 1–3 comorbidities; More than 3 comorbidities |
| **Organizational attributes** | | | |
| 12 | | Mandate | Local mandate; Regional mandate |
| 13 | | Academic affiliation | University hospital; Community hospital |
| 14 | | Geographic location | Urban; Semi-rural; Rural |
| 15 | | Cancer team size and diversity | Cancer team with 8 professionals from different disciplines and more = large;  Fewer than 8 = small) |
| * Emotional well-being (6 items) from the Health Education Impact Questionnaire (heiQ)  ** In Quebec, Business college/CEGEP are post-secondary institutions that provide pre-university education (2 years) or specialized vocational programs (3 years). | | | |

# References
