# Supplementary material for: Determinants of patient-reported experience of cancer services responsiveness
Source: BMC Health Serv Res. 2015 Sep 28;15:425. doi: 10.1186/s12913-015-1104-9 (PMC4587918; doi:10.1186/s12913-015-1104-9)
Supplement: Additional file 2: — Effects of patient characteristics and organizational attributes on cancer services responsiveness (univariate logistic regression). Additional file 2 presents the results for the univariate logistic regression to identify the effects of patient characteristics and organizational attributes on cancer services responsiveness subscales: prompt access to care (PAC), person-centred response (PCR), quality of patient-provider communication (COM), and quality of care environment (QCE) and overall CSR. (DOCX 22 kb) [file 12913_2015_1104_MOESM2_ESM.docx]

# Additional File 2: Effects of patient characteristics and organisational attributes on cancer services responsiveness (univariate logistic regression)

Independent variables included in the univariate logistic regression analysis are those that were found to be significantly correlated (p <0.05) with the overall CSR mean score following the Spearman analysis. Table 1 presents the results for the univariate logistic regression to identify the effects of patient characteristics and organizational attributes on cancer services responsiveness subscales: prompt access to care (PAC), person-centred response (PCR), quality of patient–provider communication (COM), and quality of care environment (QCE) and overall CSR. Five patient characteristics were significantly associated with likelihood of a positive rating of overall responsiveness: self-assessed health status, age, gender, education level and perceived emotional well-being. Self-assessed health status, emotional well-being and gender were associated with most of the responsiveness subscales. Academic affiliation and the geographic location (rural) were the two organisational variables associated with the likelihood of positive rating of overall responsiveness. Geographic location (rural) is the only organisational variable associated with most responsiveness subscales.

## Table S1. Univariate logistic regression: significant effects of patient characteristics and organizational attributes on cancer services responsiveness

|  |  | | **Patient reported experience measure** | | | | | | | | | |
| --- | --- | --- | --- | --- | --- | --- | --- | --- | --- | --- | --- | --- |
|  |  | | **PAC** | | | **PCR** | | | **COM** | **QCE** | | **CSR** |
|  |  | | OR [CI95%] | | | OR [CI95%] | | | OR [CI95%] | OR [CI95%] | | OR [CI95%] |
| **Patient characteristics** | | |  | | |  | | |  |  | |  |
| Self-assessed health status ^a^ | Good | | 1.38* | | | 1.79* | | | 1.53* | 1.63* | | 1.57* |
|  |  | | [1.06 - 1.78] | | | [1.40 - 2.29] | | | [1.22 - 1.91] | [1.27 - 2.09] | | [1.26 - 1.97] |
| Age ^b^ : | 50–69 | | 1.21 | | | 1.14 | | | 1.37* | 1.49* | | 1.39* |
|  |  | | [0.85 - 1.73] | | | [0.82 - 1.59] | | | [1.01 - 1.87] | [1.08 - 2.06] | | [1.03 - 1.88] |
|  | 70 years and older | | 1.32 | | | 1.37 | | | 1.62* | 2.76* | | 1.68* |
|  |  |  | [0.86 - 2.01] | | | [0.92 - 2.05] | | | [1.13 - 2.33] | [1.83 - 4.18] | | [1.17 - 2.41] |
| Gender ^c^ | Men | | 1.54* | | | 1.32* | | | 1.20 | 1.29* | | 1.34* |
|  |  | | [1.18 - 2.00] | | | [1.02 - 1.70] | | | [0.95 - 1.52] | [1.001 - 1.67] | | [1.07 - 1.69] |
| Education ^d^ | High school and less | | 1.26 | | | 1.51* | | | 1.70* | 1.39* | | 1.53* |
|  |  |  | [0.97 - 1.66] | | | [1.18 - 1.93] | | | [1.35 - 2.14] | [1.08 - 1.79] | | [1.22 - 1.92] |
| Emotional well-being ^e^ | Good | | 1.46* | | | 1.25 | | | 1.21 | 1.66* | | 1.29* |
|  |  | | [1.13 - 1.90] | | | [0.98 - 1.59] | | | [0.96 - 1.51] | [1.29 - 2.13] | | [1.03 - 1.61] |
| **Organizational attributes** | | |  | | |  | | |  |  | |  |
| Mandate ^f^ | Local | | 1.15 | | | 0.97 | | | 1.08 | 1.59* | | 1.18 |
|  |  | | [0.89 - 1.49] | | | [0.77 - 1.24] | | | [0.86 - 1.35] | [1.24 - 2.03] | | [0.95 - 1.47] |
| Academic affiliation ^g^ | Yes | | 0.95 | | | 0.75* | | | 0.70* | 0.65* | | 0.65* |
|  |  | | [0.73 - 1.23] | | | [0.58 - 0.97] | | | [0.55 - 0.88] | [0.50 - 0.84] | | [0.52 - 0.82] |
| Team size ^h^ | Large | | 0.55* | | | 1.19 | | | 0.94 | 0.60* | | 0.85 |
|  |  | | [0.42 - 0.71] | | | [0.93 - 1.51] | | | [0.75 - 1.17] | [0.47 - 0.77] | | [0.68 - 1.05] |
| Geographic location ^i^ | Semi-rural | | 1.01 | | | 1.16 | | | 1.08 | 0.82 | | 1.19 |
|  |  | | [0.71 - 1.44] | | | [0.84 - 1.60] | | | [0.81 - 1.45] | [0.60 - 1.11] | | [0.89 - 1.59] |
|  | Rural | | 2.17* | | | 1.14 | | | 1.50* | 1.56* | | 1.63* |
|  |  | | [1.62 - 2.90] | | | [0.87 - 1.50] | | | [1.16 - 1.94] | [1.16 - 2.08] | | [1.27 - 2.10] |
| Results show odds ratios (OR) and confidence intervals [CI] 95% when both patient characteristics and organizational attributes are in the model. | | | | | | | | | | | | |
| * p <0.05 | |  | |  |  | | |  | |  |  | |
| ^a^ Reference category (1) = Bad | | | |  | | | ^f^ Reference category (1) = Regional | | | |  | |
| ^b^ Reference category (1) = 18–49 | | | |  | | | ^g^ Reference category (1) = No | | | |  | |
| ^c^ Reference category (1) = Women | | | |  | | | ^h^ Reference category (1) = Small < 8 | | | |  | |
| ^d^ Reference category (1) = College and more | | | | | | | ^i^ Reference category (1) = Urban | | | |  | |
| ^e^ Reference category (1) = Negative perception | | | | |  | | |  | |  |  | |
